# Dietary practice and associated factors among type 2 diabetic patients in Felege Hiwot Regional Referral Hospital, Bahir Dar, Ethiopia

**DOI:** 10.1186/s13104-018-3531-2

**Published:** 2018-07-03

**Authors:** Yeshalem Mulugeta Demilew, Abiot Tefera Alem, Amanu Aragaw Emiru

**Affiliations:** 1 School of Public Health, College of Medicine and Health Sciences, Bahir Dar University, P.O.Box 79, Bahir Dar, Ethiopia; 2 School of Medicine, College of Medicine and Health Sciences, Bahir Dar University, P.O.Box 79, Bahir Dar, Ethiopia

**Keywords:** Dietary practice, Type 2 diabetes, Patients, Ethiopia

## Abstract

**Objective:**

Even if patient’s dietary practice is a gold standard measure to manage type 2 diabetes, there is a limited study in the area. Therefore, the objective of this study was to assess dietary practice and associated factors among type 2 diabetic patients.

**Result:**

The study revealed that only 35.9% of the patients had good dietary practice. Attending above primary education [AOR = 1.9, 95% CI (1.1, 3.2)], having family support [AOR = 2.6, 95% CI (1.6, 4.2)], and receiving nutrition education [AOR = 2.5, 95% CI (1.5, 4.2)] were independent predictors for good dietary practice. Thus, the findings indicate the need to improve a method of nutrition education both for the patients and their families. Moreover, the government needs to improve literacy rate of citizens.

## Introduction

Diabetes mellitus (DM), a set of metabolic disorders, is characterized by persistent high blood glucose level because of errors in insulin secretion, its action, or both [1, 2]. Diabetes in the general population can be of two types, namely type 1, and type 2 DM [2]. Type 1 DM makes up 5–10% of subjects and is more common among children and adolescents. On the other hand, more than 90–95% of patients belong to type 2 diabetes, and most of them are adults [3].

Type 2 diabetes mellitus (DM) is among the leading causes of illness and death in the world. For example, in 2011 there were 366 million type 2 DM cases and the number is anticipated to rise above 552 million in 2030 [4]. In Ethiopia, the prevalence of DM was estimated to be 6.5% [5, 6].

Uncontrolled diabetes predisposes patients to develop complications such as stroke, cardiovascular diseases, nephropathy, neuropathy, and retinopathy. The situation in Ethiopia is not different from the rest of the world. Gebre, for instance, pointed out that the magnitude of DM related hypertension, retinopathy, neuropathy, and DM foot ulcer were rising in the country [7]. The same study also showed a higher rate of infectious diseases such as urinary tract infection, hepatitis, and tuberculosis among DM patients than the general population.

Many studies indicated the role of appropriate dietary intake to control type 2 DM. Consumption of adequate amount of energy and nutrients is important to decrease high blood glucose level and to slow progress of the disease [8, 9]. However, dietary practices of type 2 diabetes patients differ across and within countries. For example, good dietary practice ranges from 3.1% in Brazil to 56% in Oman [10–15]. In Addis Ababa, Ethiopia 48.6% of type 2 diabetes patients had good dietary practice [16, 17]. Though the dietary practice is culture and context specific, there is limited study about the dietary practice of type 2 DM patients in the study area. Therefore, the present study was designed to assess the dietary practice and associated factors among type 2 DM patients who had follow up at Felege Hiwot Referral Hospital.

## Main text

### Methods

#### Study setting

The study was carried out in Felege Hiwot Referral Hospital. The hospital was found in Bahir Dar City, situated 565 km away from Addis Ababa, Ethiopia in the Northwest direction. During the time of data collection, there were 5629 type 2 DM patients who had follow-up in the hospital.

#### Study design and study population

Institution based cross-sectional study was conducted among type 2 DM patients. Type 2 DM patients who had follow-up visit in the diabetes referral clinic during the study period were the study population.

#### Sample size, sampling procedure and data collection

A sample size of 423 was calculated using single population proportion formula with assumption of: 95% confidence level, proportion of good dietary practice among type 2 DM patients 48.6% [18], 5% margin of error, and 10% non-response rate. Systematic sampling technique was used to select the study participants. The K-value (the interval) was calculated by dividing total number of type 2 DM patients (5629) from diabetes referral clinic registration log book to the calculated sample size. Accordingly, every 13 patients were selected, with the first sample chosen by lottery method. Interviewer-administered structured questionnaire, adapted from previous similar literature [18–20], was used to collect the data.

Two experienced nurses and one laboratory technician were recruited for data collection. While the nurses were responsible for interviewing the patients, the role of the laboratory technician was for performing blood glucose level. In addition, a public health professional was assigned as their supervisor.

Height and weight of patients were measured. During weight measurement, each participant was subjected to wear light clothes. Their weight was measured to the nearest 0.1 kg using weighing scale (Sica Germany). Similarly, height was measured to the nearest 0.1 cm using stadiometer. During the measurement, each participant stood on the measuring board without shoes, considering the normal anatomical position, while heels, buttocks, shoulders, and back of the head of patients touching the board. For both height and weight two readings were recorded, and the computed average was used in the analysis. The ratio of weight in kilograms to the square of height in meters was used to determine body mass index.

The blood sample was collected from each participant. Trained laboratory technician collect the blood sample by finger puncture, following aseptic technique. The sample was collected in the morning before participants took their breakfast (after 8 h of fasting). The blood samples were immediately taken to the hospital laboratory for chemistry analyses. Fasting blood glucose test was carried out using 902 Automatic Analyzer with Roche/Hitachi kit.

The dietary practice was assessed using the modified form of the fourteen item scales taken from previous literature [18, 20]. The items were focusing on the short and long term dietary plan of patients; their attitude towards preparing diabetes diet; selection of foods items in their daily meal; and pattern of food intake within a day. The items had “Yes” or “No” response. Value 1 was given for the “Yes” response and 0 for the “No” response. First, the median value of the fourteen items for each patient was computed to classify the respondents’ dietary practice. Then, patients who scored above the median value for the response were classified as having good dietary practice and poor dietary practice otherwise.

#### Data quality control

Two days training was given for data collectors and supervisors. The pre-test was done on 5% of the sample size. The supervisor and investigators closely supervised data collection technique on daily basis. Weight measuring scale was adjusted by setting it to zero before weighing every participant.

#### Data processing and analysis

The data entry and analysis was done using SPSS version 20 for software. The dietary practice of each patient was computed from composite variables. Bivariate analysis was done and explanatory variables with P value ≤ 0.2 were taken to the multivariable logistic regression analysis to control confounders. *P* value < 0.05 at 95% confidence interval was taken as statistically significant in the final model.

### Ethical consideration

Ethical Review Board of Bahir Dar University approved this research. The hospital administrator also gave letter of permission to undertake this study in the hospital. Then, study participants gave verbal consent to participate in the study. The research protocol approved by the IRB clearly defined the reasons for verbal consent. Informed Verbal consent was obtained because the study imposes minimal risk on study participants. Personal identifiers were excluded from the data collection form to maintain privacy and confidentiality throughout the study period.

### Results

From the total of 423 type 2 diabetes mellitus patients enrolled in this study, 401 of them gave complete information, with a response rate of 94.8 percent. The mean (± SD) age of type 2 DM patients was 56.05 ± (9.18) years with age range of 40–80 years. Above half, (52.1%) of the respondents were females. Majority of patients were from Amhara ethnic group (95.3%) and Orthodox Christian followers (87.5%). Nearly three-fourth of the participants were urban residents (73.3%) and married (72.8%). More than three in ten (63.6%) of the respondents had no formal education (Table 1).

**Table 1:** Socio-demographic characteristics of type 2 diabetic patients at Felege Hiwot Referral Hospital, Bahir Dar, Ethiopia, 2016, (n = 401)

Variable	Frequency	Percentage (%)
Sex		
Female	209	52.1
Male	192	47.9
Age in year		
40–60	289	72.1
> 60	112	27.9
Religion		
Orthodox	351	87.5
Muslim	50	12.5
Ethnicity		
Amhara	382	95.3
Agew	13	3.2
Tigre	6	1.5
Educational status		
Have no formal education	255	63.6
Primary education	48	12.0
Secondary education	44	11.0
Above secondary education	54	13.4
Occupational status		
Housewife	111	27.7
Farmer	79	19.7
Retired	73	18.2
Government employee	67	16.7
Merchant	44	11.0
Daily laborer	27	6.7
Marital status		
Married	292	72.8
Widowed	40	10.0
Single	37	9.2
Divorced	32	8.0
Residence		
Urban	294	73.3
Rural	107	26.7

#### Health status and self-care practice of type 2 diabetes patients

Duration of the disease for 60.8% of the respondents was ≤ 5 years. More than one in three (37.4%) respondents had other chronic diseases co-morbidities, mainly hypertension (29.4%) and heart diseases (4.5%). More than one-third (34.9%) of the patients were overweight/obese. Only 7% of the participants were measuring their blood glucose level at their home, and 24.2% got nutrition education from health professionals.

#### Dietary practice

In this study about 35.9% [95% CI (31.0, 41.0), P-value, 0.024] of type 2 diabetes patients had good dietary practice (Table 2). More males (40.1%) compared to females (32.1%) had good dietary practice. Besides, urban residents had better dietary practice than rural dwellers (39.5% vs 26.2%).

**Table 2:** Dietary practice of type 2 diabetic patients at Felege Hiwot Referral Hospital, Bahir Dar, Ethiopia, 2016, (n = 401)

Variable	Frequency	Percentage (%)
Plan the meals she/he eat ahead		
Yes	331	82.5
No	70	17.5
Took meal based on dietary plan yesterday		
Yes	264	65.8
No	137	34.2
Took meal based on dietary plan over the past 2 weeks		
Yes	185	46.1
No	216	53.9
Always eat based on dietary plan		
Yes	150	37.4
No	251	62.6
Always eat based on dietary plan, even when she/he feels her/his blood glucose level is controlled		
Yes	163	40.6
No	238	59.4
Never feel hassled to stick on dietary plan		
Yes	210	52.4
No	191	47.6
Have no feelings of dietary deprivation		
Yes	225	56.1
No	176	43.9
Follow flexible eating plan		
Yes	147	36.7
No	254	63.3
Eat fruits daily		
Yes	37	9.2
No	364	90.8
Eat vegetables daily		
Yes	45	11.2
No	356	88.8
Cut down butter intake		
Yes	265	66.1
No	136	33.9
Cut down fat intake		
Yes	318	79.3
No	81	20.7
Cut down sweet and soft drink intake		
Yes	255	63.6
No	146	36.4
Always follow regular meal time		
Yes	128	31.9
No	273	68.1
Overall practice		
Good	144	35.9
Poor	257	64.1

#### Factors affecting the dietary practice of type 2 diabetic patients

The bivariate logistic regression analysis showed that sex, educational status, nutrition education on diabetes diet, availability of fruits, availability of vegetables, family support and awareness on diabetes diet were statistically associated with the dietary practice of type 2 diabetic patients.

From the multivariable logistic regression analysis, type 2 diabetic patients who get counseling on diet were 2.5 times more likely to have better practice than their counterparts [AOR = 2.5, 95% CI (1.5, 4.2)]. Likewise, patients who attended secondary education and above were 1.9 times more likely to have good dietary practice than those who attend less than secondary education [AOR = 1.9, 95% CI (1.1, 3.2)]. The odds of having good dietary practice was 2.6 times higher among respondents who had awareness on diabetes diet than patients who had no awareness [AOR = 2.6, 95% CI (1.5, 4.6)]. Family support also showed association with dietary practice. In this aspect, Participants who had family support were 2.6 times more likely to have good dietary practice than patients who did not have family support [AOR = 2.6, 95% CI (1.6, 4.2)] (Table 3).

**Table 3:** Factors associated with dietary practice of type 2 diabetic patients at Felege Hiwot Referral Hospital, Bahir Dar, Ethiopia, 2016

Variable	Dietary practice		COR (95% CI)	AOR (95% CI)
	Good practice	Poor practice		
Get nutrition education on diabetes diet at hospital				
Yes	48 (49.5)	49 (50.5)	2.1 (1.3, 3.3)	2.5 (1.5, 4.2)**
No	96 (31.8)	206 (68.2)	1.00	1.00
Availability of fruits				
Yes	44 (46.3)	51 (54.7)	1.7 (1.1, 2.8)	
No	100 (32.7)	206 (67.3)	1.00	
Availability of vegetables				
Yes	37 (46.8)	42 (53.2)	1.7 (1.1, 2.9)	
No	107 (33.2)	215 (66.8)	1.00	
Have family support				
Yes	61 (51.3)	58 (48.7)	2.5 (1.6, 3.9)	2.6 (1.6, 4.2)**
No	83 (29.4)	199 (70.6)	1.00	1.00
Awareness on diabetes diet				
Yes	120 (40.8)	174 (59.2)	2.3 (1.4, 3.9)	2.6 (1.5, 4.6)**
No	24 (22.4)	83 (77.6)	1.00	1.00
Educational status				
Less than secondary	92 (30.4)	211 (69.6)	1.00	1.00
Secondary and above	52 (53.1)	46 (46.9)	2.1 (1.3, 3.4)	1.9 (1.1, 3.2)**
Thinking of low cost of food				
Yes	109 (38.1)	177 (61.9)	1.4 (0.8, 2.2)	
No	35 (30.4)	80 (69.6)	1.00	
Sex				
Male	77 (40.1)	115 (59.9)	1.4 (0.9, 2.1)	
Female	67 (32.1)	142 (67.9)	1.00	
Residence				
Urban	116 (39.5)	178 (60.5)	1.8 (1.1, 3.0)	
Rural	28 (26.2)	79 (73.8)	1.00	
Age (years)				
40–60	107 (37.0)	182 (63.0)	1.1 (0.7, 1.8)	
> 60	37 (33.0)	75 (67.0)	1.00	
Marital status				
Married	101 (34.6)	191 (65.4)	0.8 (0.5, 1.2)	
Single/divorced/widowed	43 (39.4)	66 (60.6)	1.00	

*COR* crude odds ratio, *AOR* adjusted odds ratio, *C/I* confidence interval

** P-value < 0.01

### Discussion

In this study only 35.9% [95% CI (31.0, 41.0), P-value, 0.024] of the respondents had appropriate dietary practice. This practice is better than the study finding in Tikur Anbessa Specialized Hospital, Addis Ababa, Ethiopia (22.2%) [21]. The difference might be due to the time gap between studies; currently, more nutrition education is given on diabetes diet than do the earlier. Moreover, there is better internet access and media coverage now than in the past, which might contribute for improving knowledge on diabetes diet.

On the other hand, this practice is lower than study findings in Yekatit 12 Hospital, Addis Ababa, Ethiopia (48.6%) [22] and Botswana (62.8%) [23]. This discrepancy might be due to the difference in study settings and study subjects; this study was done among urban and rural residents with the low level of education whereas the former studies were done among urban residents with better educational status.

Patients who got nutrition education on diabetes diet were more likely to have good dietary practice than their counterparts. This finding is similar with the study findings in Ethiopia [24], Iran [25], and Nepal [26]. This may be due to the fact that nutrition education changes dietary behavior.

Educational status of respondents had an association with their dietary practice. This is in line with study findings in Addis Ababa, Ethiopia [21, 22], Iran [25] and Bahrain [27]. The correlation between higher educational attainment of respondents and appropriate dietary practice might be explained by the fact that patients with better education have better access to information from books, leaflets, newspaper and social media than uneducated patients. Additionally, educated participants could better understand nutrition education given by professionals or through mass media than uneducated people.

Having awareness of diabetes diet was an important predictor of good dietary practice. This is in agreement with the study finding in Nepal [26]. When people have awareness on the benefit of having diabetes diet plan they are more likely to adopt and maintain the newly adopted behavior [28, 29].

Family support was also another predictor of appropriate dietary practice. This is in agreement with the study finding in Bahrain [27]. Positive family support is the means of promoting preventive measures like good dietary practice and other diabetes self-care practices [30, 31].

### Conclusion and recommendation

In this study, the dietary practice of type 2 diabetes patients was poor. Factors associated with good dietary practice were getting nutrition education on diabetes diet management, attending secondary school and above, and having awareness of diabetes diet and family support. Thus, findings recommend the need to give context based nutrition education to patients and families. Moreover, the government needs to work more in improving the literacy rate of citizens.

## Limitation

Assessing the level of practice using self-reported dietary practice might introduce social desirability bias. However, to minimize this bias detail explanation was provided about the aim of the study.

The result need to be used with caution as some of the variables (such as availability of fruits and vegetables) did not show association in our final model which otherwise could have contribution to dietary practice of diabetes patients. Thus, we recommend future studies with large sample size.

## Abbreviations

AOR: adjusted odds ratio
CI: confidence interval
COR: crude odds ratio
DM: diabetes mellitus
